# Quantifying “local food” online and social media in the United States for 2018–2021

**DOI:** 10.1186/s40066-022-00397-y

**Published:** 2022-12-16

**Authors:** Jinho Jung, Jingjing Tao, Nicole Olynk Widmar

**Affiliations:** Department of Agricultural Economics, Purdue University, 403 West State Street, West Lafayette, IN 47907 USA

**Keywords:** Food security, Local food, Public perceptions, Social media data

## Abstract

**Background:**

Interest in local food has been growing, driven by increased attention from consumers, supporting policies, and interest in offering supply by local producers. Nonetheless, a definition of “local food” remains elusive, varying with purposes, geographies, and perceptions. This study quantifies online media mentioning local food in 2018–2021 using online and social media listening and analytics. In addition, a sub-search devoted to local food security and access was conducted due to a high proportion of mentions devoted to food security in the initial search. Variations in mentions and net sentiment quantified for individual US states are also presented.

**Results:**

The local food pantry sub-search arose after finding a large share of the general local food media was referencing local food access rather than production or other topics. The interest in local food access was more apparent during crises periods, such as the COVID-19 pandemic, during which even a larger portion of mentions are devoted to the local food pantry sub-search topic. Mentions quantified from the sub-search are mostly expressing concerns about worsened food insecurity during the pandemic and encouraging others to do things like donate food to local pantries.

**Conclusions:**

Online and social media can play an important role towards active communication in local communities on topics, such as food availability and access. In addition, online media can facilitate more efficient emergency management.

## Introduction

Food and farm businesses, generally known as industries with thin profit margins, generally seek to scale up production (volume) to be efficient and profitable. With all the structural changes in agricultural production industries in combination with the development of national and global supply chains, food that had been provided from local and/or regional farms in the past has moved to being procured from areas with the lowest cost of production [1, 2]. Simultaneously, many operations have taken on strategies of selling differentiated products to specialty retailers, food co-ops, and food service companies to seek premiums and bigger margins [3, 4]. This differentiation has contributed to a resurgence of consumers’ interest in who produces food, how it is grown, and how far food travels. These growing interests in food production, among many other societal considerations, have induced consumers to consider purchasing food directly from farmers [5–8].

The increase in producers’ interest in the local food system may be related to a rise in demand, but it has also been a result of policy promoting local food systems. For example, the number of farms engaged in direct-to-consumer (DTC) sales, such as farmers market, Community Supported Agriculture (CSA), and other forms^1^ increased by 5.5% from 136,817 to 144,530 farms between 2007 and 2012 [9], reaching at 167,009 in 2015 [10]. Thus we have seen an increase in the value of local food sales through DTC, retailers, institutions, and intermediaries [11]. Local food systems have been a subject of policy debate in federal, state, and local governments, because local foods have been discussed in the contexts of sustainability, rural economy, and strengthening agricultural producers and markets [5]. Consistent with USDA priorities [11], a growing number of government programs^2^ and state and local policies^3^ for local food initiatives [11, 12] have been introduced.

The definition of local food remains unclear. Some people perceive it of a more environmentally and climate friendly alternative. Others see it a safer, fresher, and healthier products than the imported [5]. The lack of a universal definition may make it difficult for consumers to identify local products and to guarantee that products labelled as ‘local food’ meet their expectations [5]. Explored in previous academic studies, the definitions range from distance, political boundaries, to specialty criteria [5]. That being said, defining the term is crucial to ensure that people perceive it in a consensus view, economic activities can be well measured and research objectives can be clearly targeted. When it comes to how people define local food, previous studies focus on how researchers and consumers perceive local food and define “local” [11, 12, 16–18]. In a physical distance, the 2008 Farm Act defines local food as products purchased and consumed within 400 miles from its origin [12] or within the boundaries of states, where they are grown and sold [11]. According to Adams and Adams [19], some consumers perceive “localness” based on local ownership of the farms or associate local food with natural, organic and other specialty foods. People perceive that an attribute of being locally grown is important in public food consideration [17]. According to Schupp [20], the local food movement is correlated with the presence of farmers’ markets. Furthermore, local food systems in rural places are unique compared to urban areas, in terms of local production flows, social, ecological, and economic benefits, and opportunities and obstacles [21].

### Local food systems and food security

The topic of local foods has been examined in its own right, but also as a part of discussions about sustainable agriculture [22, 23], food accessibility [24], and integrated studies of community and human well-being [25, 26]. Studies under the umbrella of sustainable agriculture seek to integrate healthy environment, food security, and/or economic profitability. Sustainable agriculture is talked about as trying to satisfy needs for nutritious and healthy foods, address food security, provide information about the supply chain for improved food safety, and enhance income and quality of life of farm families, while simultaneously investigating the impacts of production on the environment. Research topics such as environmentally friendly production practices (conventional and organic), perception of local food, carbon footprint with reduced transportation, food security, the resilience of local food systems with local food bank/pantry, food waste, etc. contribute to understanding of sustainability in this context [27].

Studies on local food are numerous about consumer’s perceptions, purchasing behavior, and perspectives [11, 12, 16–18, 21]. Existing work on food security in local communities is largely about the role that local food banks plays in improving nutritional intake and health status of the vulnerable, reducing food insecurity, and more recently alleviating negative impacts of social distancing on food insecurity under the COVID-19 pandemic [28–31].

This study aims to examine public perceptions on local food using online and social media listening and analytics to amass a novel data set. The use of online media offers a unique approach which adds to the often survey-based data commonly described in the literature. State-specific data facilitates understanding of social media volume and sentiment surrounding the local food topic and allows for geographical, in addition to temporal, analyses. Concepts and usefulness of social media listening will be introduced and described in the next section before then describing methodological details of how social media listening was used in this study.

### Online and social media data in markets

Social media is being used as a channel for marketing and public relations [32] and public communication or campaigns by government and non-profit organizations [33]. Given the increase in online media use, it is important to study how individuals react to, share, amplify, or create marketing, campaign, and emergency response materials. This has led to the advent of social listening. A benefit that social listening can provide, while traditional surveys cannot is that mentions or posts arise without prompt or questioning offering the potential for related topics or unforeseen insights that may not have been considered when generating survey questions.

Social media listening enables searches of online and social media and quantifies sentiment expressed on online platforms [34], such as Twitter, forums, blogs, comments and news. Social listening allows exploration of unprompted media [35]. Along with the provision of interactive space, an important advantage of social media over traditional communication channels is its speed. Social media makes it possible to create online posts in real-time, react to online content immediately and disseminate information quickly [36]. In addition, the rapidity of communication on social media may enable governments to effectively manage certain crises, such as natural disasters [37, 50, 51] or food recalls [38, 39], via social media communication.

The purpose of this study is to quantify the volumes, sentiment, and topics associated with online and social media about local food in the United States in 2018–2021. In addition, since main local food items, their related situation, demographics, and interesting topics may vary by region, the volumes and sentiment of search results will also be examined by state. Understanding how local food is discussed generally in online and social media spaces may inform public policy and decision making by various agencies. The remainder of this article as follow, the next section describes how social media listening is utilized for this study in more detailed technical terms, results and its discussion will be presented, then moving onto conclusion section for closing and summarizing remarks.

## Materials and methods

Online and social media is an underused data source for agricultural and food markets [40]. Nowadays, numerous online databases, web search engines, and social listening analytics are utilized to facilitate data collection and analysis. For example, LexisNexis [41] offers government agencies and universities news, business sources, and searching capacities [42]. More recently, other social listening platforms such as Brand 24, Brandwatch, and Netbase have been developed.

The Netbase platform, which is a leader in social media analytics, social listening, and intelligence among these tools was employed for data collection in this study with inclusionary and exclusionary search terms and keywords identified by researchers. Netbase provides volumes of mentions (sentences retrieved from posts) and posts (entire discussions from a social media space), sentiment (through a natural language processing (NLP)), top terms and hashtags, and other information over a specified timeline [43, 44]. To parametrize the searches of online and social media researchers developed search terms and keywords related to the local food topic. The search was conducted over the time period of 2018 through 2021. This 4-year timeframe was selected to detect and examine seasonal patterns associated with local food mentions and is similar to other previous social listening analyses utilizing the Netbase platform [37–40]. The study period encompasses pre and during COVID-19 time periods, allowing comparisons of search results during the pandemic era when food supply chains were stressed and interest in the local provision of food items was hypothesized to rise.

While searches are technologically possible across all languages, there are limitations of understanding caused by slang, shorthand, sarcasm, and cultural context. Thus this search was limited geographically to within the US and searches were conducted in English exclusively. Numbers of posts, mentions, and net sentiment was collected at daily granularity for the time periods 2018–2021 on January 7th, 2022. Top mentions and the state-specific metrics were collected annually for each of 4 years studied.

Two stages of data collection were conducted. First, a search with only primary search terms related to local food and a new trendy food term [45], *locavore*^4^, adopted by the Oxford American Dictionary in 2007, was conducted. The primary search terms used to parametrize this initial search included: Local food, #localfood, locavore, and #locavore with some modified with hashtags (hereafter, the general local food search). Second, the search results from the step one were filtered, or searched within, to develop a subsearch devoted to food security and access. The results from the general local food search revealed 38% of the total mentions over the 4-year period were related to local food bank/pantry (374,247 mentions out of 991,072 mentions), leading researchers to filter the data from the general local food searches based on specific terms including food bank, food pantry, food security, and food insecurity, including some of them modified with hashtags (hereafter, the local food pantry searches).

The sentiment of public media or social media conversations refers to the general view, attitude, or opinion towards a topic, event, situation, or phenomenon. The net sentiment used in this analysis is a construct to capture the positivity, negativity, and neutrality of the media returned in the searches conducted. Within social listening literature, net sentiment is a designed measurement of comparing positive and negative posts to arrive at a single numerical value. The neutral category of posts is included when calculating and reporting on analytics for top words and other data summaries but is not used in the calculation of net sentiment. The social media net sentiment score, a numeric value, was assigned with the help of Natural Language Processing (NLP) engine from the Netbase [44] platform. Net sentiments have been calculated as the result of the total percentage of positive posts minus the percentage of negative posts, which is necessarily bounded between -100% (completely negative) and + 100% (completely positive).

## Results and discussion

The study time period was from January 2018 to December 2021, which is long enough to cover several annual events related to local food, such as harvesting seasons. Of particular interest is the period that covers 2020 through 2021, during which unemployment rose alongside food prices. Many recent studies on food security have investigated the impact of COVID-19 on food security and the resilience of local food systems. Due to the pandemic and resulting economic implications, more individuals are anticipated to experience food insecurity [28, 29, 31].

**Fig. 1:**
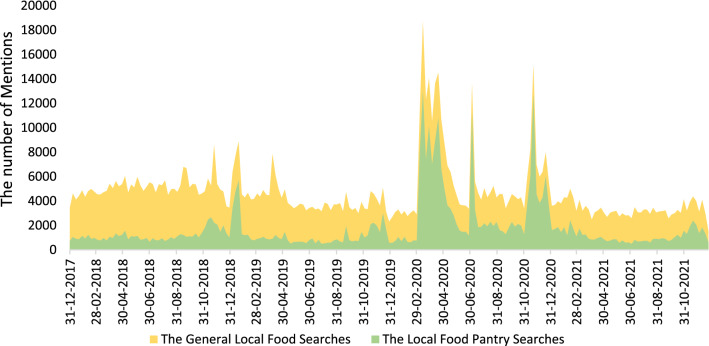
Monthly changes in the number of mentions for both the general local food searches and the local food pantry searches

Table 1 presents the top domains and sources of search results for the general local food search, the food pantry oriented subsearch, and the component of the general local food search not included in the food pantry subsearch. Twitter.com consists of the largest portion of domains and sources across all three data sets. The news was the second most popular in terms of sources. More total mentions were posted in 2020 than in other years for both searches. For the general local food searches, the number of mentions increased from 140,169 to 225,634 for domains (by 61%) and 220,644 to 329, 494 for the source (by 49%). Even larger increases were found for the local food pantry searches (42,796 to 147,584 for domains (by 245%) and 64,120 to 190,083 for sources (by 196%)). On the other hand, the number of mentions for the searches excluding local food pantries does not show a similar increase. Rather, the number of mentions decreased by 17% for domains and by 13% for sources from 2019 to 2020. Figure 1 depicts both the general local food searches and the local food pantry subsearch in the US, the US minor outlying islands, and Puerto Rico over the 4-year study period. A large portion of the total mentions were about the topic of local food banks and pantries and local food security. Overall, 38% of the total mentions about local food talk about local food bank/pantry. Studied over time, the proportion of total local food media devoted to local food pantries was 26% over pre-pandemic time periods (2018 and 2019) and 50% over pandemic time periods (2020 and 2021). Thus, a relatively large share of the total mentions of local food reference food banks, food pantries, or local food security and access. Considering the broad range of topics surrounding local foods, including supporting local economy and farmers, environmental aspects of farming, supply chain functionality, this large share devoted to food access is notable. Even over the period of June and July 2018 (Fig. 1) with a lower proportion (15%) of local food bank/pantry mentions, the term “local food bank/pantry” comes up as one of the top popular terms in addition to “donate/donation”.

**Table 1:** Top domains and sources for the general local food searches and the local food bank searches, 1 January 2018 to 31 December 2021

(a) Top domains and sources for the general local food searches								
	2018		2019		2020		2021	
	Domain or source	% of mentions	Domain or source	% of mentions	Domain or source	% of mentions	Domain or source	% of mentions
Total mentions	*n* = 266,611		*n* = 220,644		*n* = 329,494		*n* = 174,027	
Domains	*n* = 152,086		*n* = 140,169		*n* = 225,634		*n* = 85,829	
	twitter.com	75	twitter.com	77	twitter.com	96	twitter.com	91
	reddit.com	10	booking.com	8	booking.com	1	tripadvisor.com	2
	tripadvisor.com	7	reddit.com	7	tripadvisor.com	1	brisbanelocalfood.ning.com	2
	booking.com	5	tripadvisor.com	5	patch.com	1	booking.com	1
	hotels.com	1	patch.com	1	airbnb.com	1	msn.com	1
	morningagclips.com	1	ihs.jobs	1			boards.4channel.org	1
	my.jobs	1	morningagclips.com	1			medium.com	1
							morningagclips.com	1
Sources	*n* = 266,611		*n* = 220,644		*n* = 329,494		*n* = 174,027	
	Twitter	42	Twitter	48	Twitter	66	Twitter	46
	News	31	News	24	News	19	News	28
	Forums	17	Forums	16	Blogs	11	Blogs	21
	Blogs	9	Blogs	11	Forums	5	Forums	5
	Consumer Reviews	1	Consumer Reviews	1	Comments	1		

(b) Top domains and sources for the local food pantry searches								
	2018		2019		2020		2021	
	Search type	% of mentions	Search type	% of mentions	Search type	% of mentions	Search type	% of mentions
Total Mentions	*n* = 60,512		*n* = 64,120		*n* = 190,083		*n* = 59,469	
Domains	*n* = 33,008		*n* = 42,796		*n* = 147,684		*n* = 36,747	
	twitter.com	79	twitter.com	90	twitter.com	98	twitter.com	96
	reddit.com	15	reddit.com	7	patch.com	1	prnewswire.com	1
	com.babycenter.com^1^	1	patch.com	1	prnewswire.com	1	msn.com	1
	prnewswire.com	1	com.babycenter.com^1^	1			sg.finance.yahoo.com	1
	chicagotribune.com	1	prnewswire.com	1			business.ridgwayrecord.com	1
	mumsnet.com	1	biz.wdailynews.com^2^	1				
Sources	*n* = 60,512		*n* = 64,120		*n* = 190,083		*n* = 59,469	
	Twitter	43	Twitter	60	Twitter	76	Twitter	59
	News	36	News	25	News	16	News	27
	Forums	14	Forums	8	Blogs	6	Blogs	11
	Blogs	7	Blogs	7	Forums	2	Forums	3

(c) Top domains and sources for complement of the local food pantry searches (searches excluding local food bank/pantry)								
	2018		2019		2020		2021	
	Search type	% of mentions	Search type	% of mentions	Search type	% of mentions	Search type	% of mentions
Total Mentions	*n* = 206,099		*n *= 156,524		*n* = 139,421		*n* = 114,568	
Domains	*n* = 118,857		*n* = 96,767		*n* = 80,524		*n* = 51,301	
	twitter.com	73	twitter.com	70	twitter.com	90	twitter.com	86
	reddit.com	9	booking.com	11	booking.com	3	tripadvisor.com	3
	tripadvisor.com	8	tripadvisor.com	7	tripadvisor.com	2	brisbanelocalfood.ning.com	3
	booking.com	7	reddit.com	7	airbnb.com	1	booking.com	2
	hotels.com	1	ihs.jobs	1	morningag.com^3^	1	boards.4channel.org	2
	morningagclips.com	1	morningagclips.com	1	bo.4chan.org^4^	1	msn.com	1
Sources	*n* = 206,099		*n* = 156,524		*n* = 139,421		*n* = 114,568	
	Twitter	43	Twitter	44	Twitter	52	Twitter	39
	News	29	News	24	News	23	News	29
	Forums	18	Forums	19	Blogs	18	Blogs	26
	Blogs	9	Blogs	12	Forums	7	Forums	5
	Consumer reviews	1	Consumer reviews	1				

*n* is the total number of mentions for domains and sources, respectively. Due to the length of some domain names and limitation of the space for the table, full names of those with long domain names are put in note. Due to the length of some domain names and limitation of the space for the table, full names of those with long domain names are put in note

^1^community.babycenter.com

^2^business.wapakdailynews.com

^3^morningagclips.com

^4^boards.4channel.org

Given the collective concern about food supply chain functionality during the COVID-19 pandemic, and especially in 2020 when food systems were under acute stress [46–49], the use of online media to study public policy seems fitting and in line with previous literature. Many researchers have found positive impacts of using social media for response, recovery, and risk coordination. For example, Yin et al. [50] presented how social media platforms provide a great source of reliable details about real-life events and deliver useful insights into time-critical scenarios for responses by emergency officials. Velev and Zlateva [51] discussed the potential for using social media in the management of natural disasters. Jung et al. [38] suggests via social media analytics that initial reports of foodborne illness drive more public attention than actual recall announcements. Widmar et al. [40] found a decreasing amount of reaction to mosquito-associated diseases/risks even while disease risk persists and people were simultaneously expressing positive views on associated vaccine development. Widmar et al. [40] suggests online/social media analysis may potentially inform health officials of waning public focus and assist in assessing effectiveness of education campaigns to combat public health threats.

Local food and food security are community and social factors influenced by government policy and campaigns, where effective communication is critical [11, 12, 52–56]. Bertmann et al. [30] found that rural food pantry use (5.5%) was significantly higher than urban pantry use (3.7%) after COVID-19, albeit with less overall participation. Online groups were created to facilitate mutual aid, exhibiting community resilience [57]. Cross-sector collaboration, supply chains, and addressing gaps in service with respect to increased risk populations influenced the effectiveness of local approaches [58].

While there is no discernable seasonality in volume or sentiment of search or subsearch results, there are five peaks during which mentions from the food pantry subsearch accounted for a larger proportion of the total mentions (approximately 69% on average). The five peaks have common popular terms; local food bank and donating/donate. Although the peaks share common popular terms, one peak in the beginning of 2019 and four peaks after 2020 originate from different events. The peak in 2019 is based on the longest shutdown of the federal government in US history, while the other four coincide with major waves of the COVID-19 pandemic in 2020 [59]. The most popular post from the peak in January 2019 referenced a church in Alabama using its $14,000 emergency fund in addition to a $2,500 donation to buy grocery gift cards for furloughed federal workers during the shutdown as a part of community-wide support. There were both news articles [60] about community support through local food banks/pantries and research interests [61] about government employees relying on local food banks/pantries.

The four peaks in 2020 were mostly driven by social media activities during the pandemic. The popular media during those 2020 peaks were mostly encouraging people to donate food or money and to volunteer for deliveries to local food banks. For example, “…., consider donating money to your local food bank or relief organization, ……. assistance to those in need”, “If you are able, consider donating to a local food bank to help those in need……”, and “delivering groceries to a local food pantry.”. Given that social distancing prevents people from visiting not only brick-and-mortar grocery stores but also local food pantries, comments encouraging volunteers to deliver were also a part of popular posts.

Search results suggest that people were concerned about food insecurity during the pandemic. Many people were encouraging others through social media to do things like donating food or money to help neighbors mitigate food security or access concerns. The rising proportions of online media about local food being devoted to local food security activities mirrored other public statistics reflecting worsening food security situations during the same time period. According to Feeding America, requests for food assistance at food pantries were expected to increase almost by 50%, which necessitated them to spend more money on food [62, 63]. It is reported that demands for charitable food increased from 50 to 140% in the first month of the pandemic [64, 65]. In addition, 82% of food banks reported that they had a higher number of patrons nationwide than they did in the prior year [66]. Pandemic-related occurrences such as lay-offs, rising food prices, and limitations in travel to grocery stores and food pantries furthered efforts to encourage actions on social media which supports previous findings on utilization of social media for crisis management [37, 38, 40, 51, 67–69].

Considering that 85% of the low-income pantry users responded that local food pantries were helpful in improving food access over the pandemic [30], a remaining challenge is how to stock local food banks to meet the increased demand during crises. Bertmann et al. [30] emphasize the importance of donation, funding, maintenance, and preparedness for local charitable food services. Based on the findings on social media, people seem aware and participate by actively nudging others to donate food/money and volunteer. In this context mentions during the 2020 and 2021 can be regarded as a part of a social movement convincing others to do altruistic actions, while mentions of other emergencies such as natural disasters or food recalls might be more likely to serve as be immediate information sources.

Mentions rose substantially at the first wave of the pandemic, around March 2020, and then decayed for the second and third waves despite an increasing number of new cases [59]. This lessening of media attention over time, even while disasters persist, is a consistent finding with other studies, such as dissipating the volume of mentions of hurricanes [37, 70] and the lessening proportion of online media devoted to mosquitoes about the Zika virus [40]. The decay in social media activities over the pandemic waves might have been a result of posters' pandemic fatigue or the belief that need for posting might have diminished. Another possible reason is that government financial support such as stimulus checks or Pandemic Electronic Benefit Transfer (P-EBT) might have helped families procure food, lessening the acute burdens of local food banks/pantries.

**Fig. 2:**
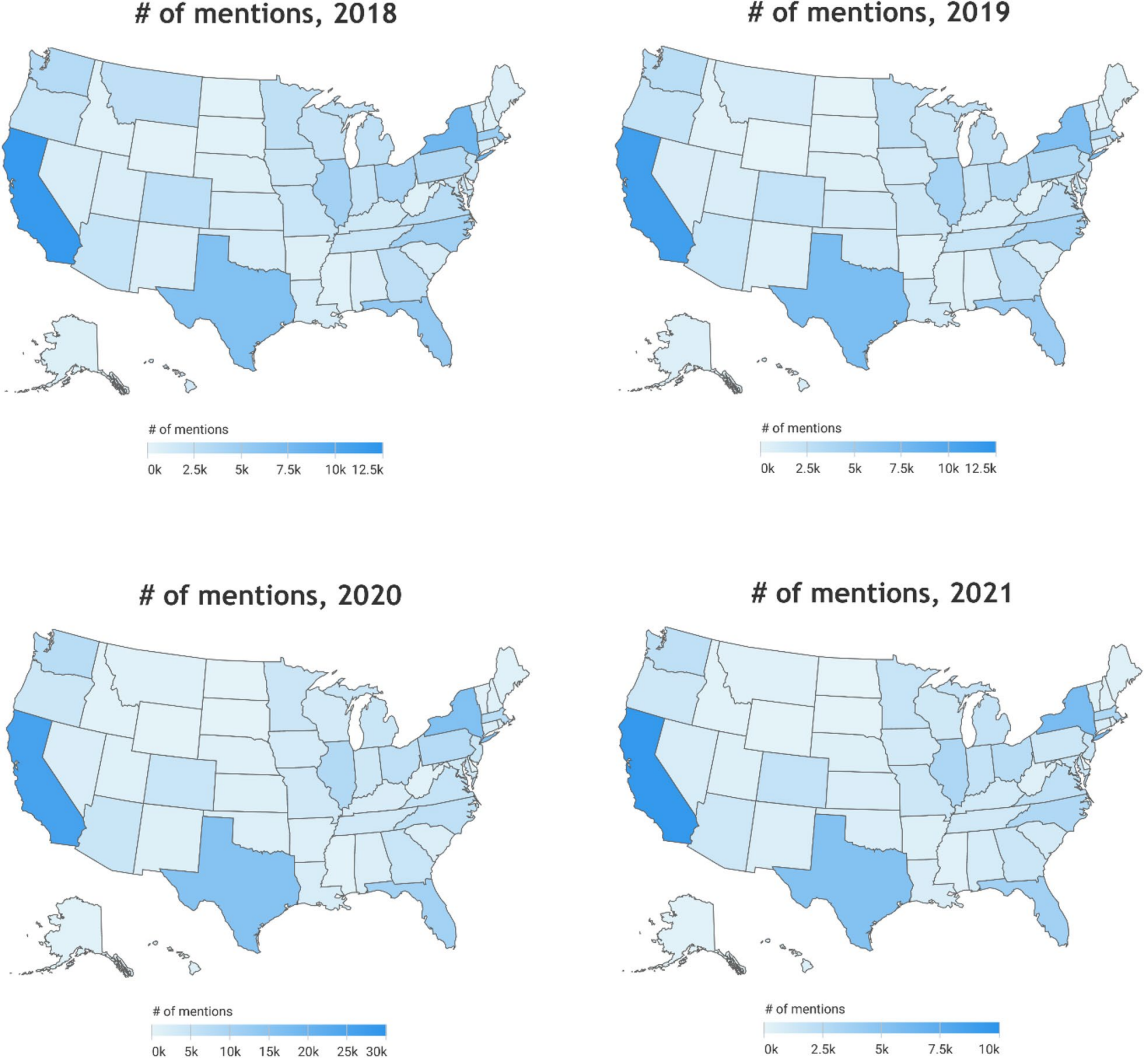
Map of the number of mentions across the US states from 2018 to 2021. Source: Authors’ generation with data collected from Netbase

**Fig. 3:**
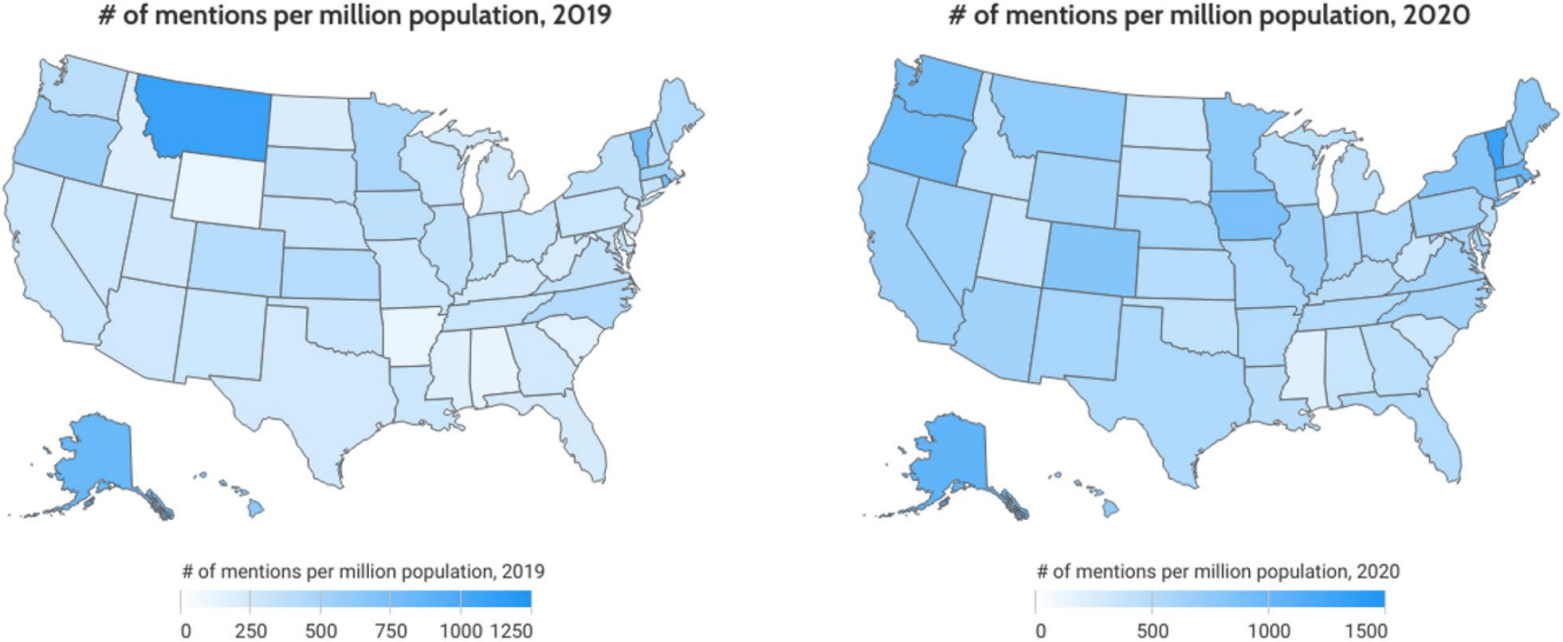
Map of the number of mentions per million population in 2019 and 2020. Source: Authors’ generation with data collected from Netbase for the number of mentions and the US Census Bureau for the US population

There is variation in the volume of mentions between US states shown in Fig. 2; however, the population variation must also be acknowledged. Figure 3 displays the volume of mentions per million people by state. As is presented, activities per capita do not vary across states. On exceptional state is Montana in 2019 reacting actively to local food pantry/bank for supporting furloughed government employees. (https://www.census.gov/quickfacts/fact/map/US/POP010220).

**Fig. 4:**
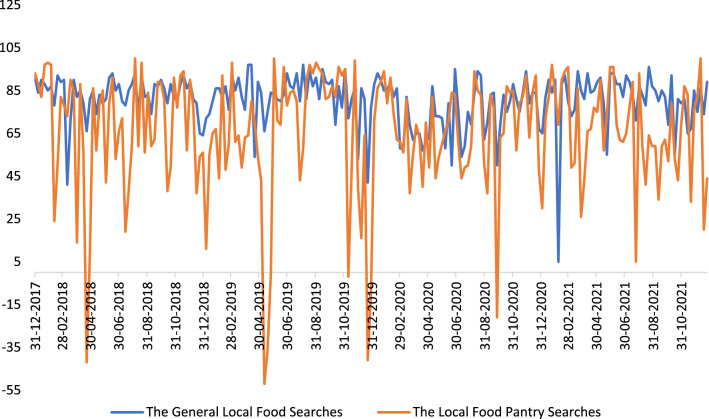
Monthly changes in net sentiment for the general local food and the local food pantry searches from January 2018 to December 2021

Figure 4 illustrates net sentiment over time for the US as a whole. Net sentiment from the local food pantry subsearch was often negative, even while the general local food search was positive. Even with findings including encouraging messages about donations to pantries, the overarching themes presented in the food pantries subsearch were more often negative than those in the general local search.

Table 2 presents the most common or top words that contributed to positive and negative sentiment in the search results, called sentiment drivers, which may provide a deeper understanding of net sentiment over the study years. Overall, the positive sentiment drivers are mostly comprised of “great local food”, “fresh food” “authentic”, and “help”. Major negative ones included “deceive consumer”, “struggle”, and “pandemic”. Note there is a shift in pattern after the year of 2019. “COVID-19”, “difficult”, “desperate”, “pressing need”, and “panic” become new key negative sentiment drivers that are not displayed in the years 2018 and 2019.

**Table 2:** Sentiment drivers for the local food pantry searches

	2018		2019		2020		2021	
	Drivers	% of mentions	Drivers	% of mentions	Drivers	% of mentions	Drivers	% of mentions
Positive	*n* = 814		*n* = 616		*n* = 2,370		*n* = 982	
	donation	11	Amazing local food bank	13	10,000 donation	21	Come through	33
	local food bank	8	Donation	13	help	17	Donation	9
	harvest	7	Help	12	Incredibly important work	13	Support	8
	meaningful local food bank economy	6	Support 46 million people	6	Local food bank	6	Local food bank	6
	local food pantry	6	Offer to help people	6	Food	4	Food	5
	work	6	Local food pantry	5	Use support right now	4	Surplus	4
	help	5	Support local food bank	4	Use help	4	Provide food for those	4
Negative	*n* = 200		*n* = 452		*n* = 804		*n* = 260	
	Diaper donation	22	Local food pantry	36	Donation	17	Local food bank	20
	Struggle to pay bill	16	Domestic violence	11	Struggle	14	Donation	17
	Local food bank	7	Donation	6	Difficult	11	Hit	10
	Local food pantry	7	Struggle to find volunteer	5	Lack of available space	8	Struggle to feed people	8
	Issue with worker	5	Face crises	5	Hit	6	Weather pandemic	7
	Support food insecure family	5	Strain	4	Coronavirus	6	Local food security	4

*n* is the total number of mentions for likes and dislikes, respectively. This is based on the actual words extracted from social media and lower letters are used for the first letter of each

**Fig. 5:**
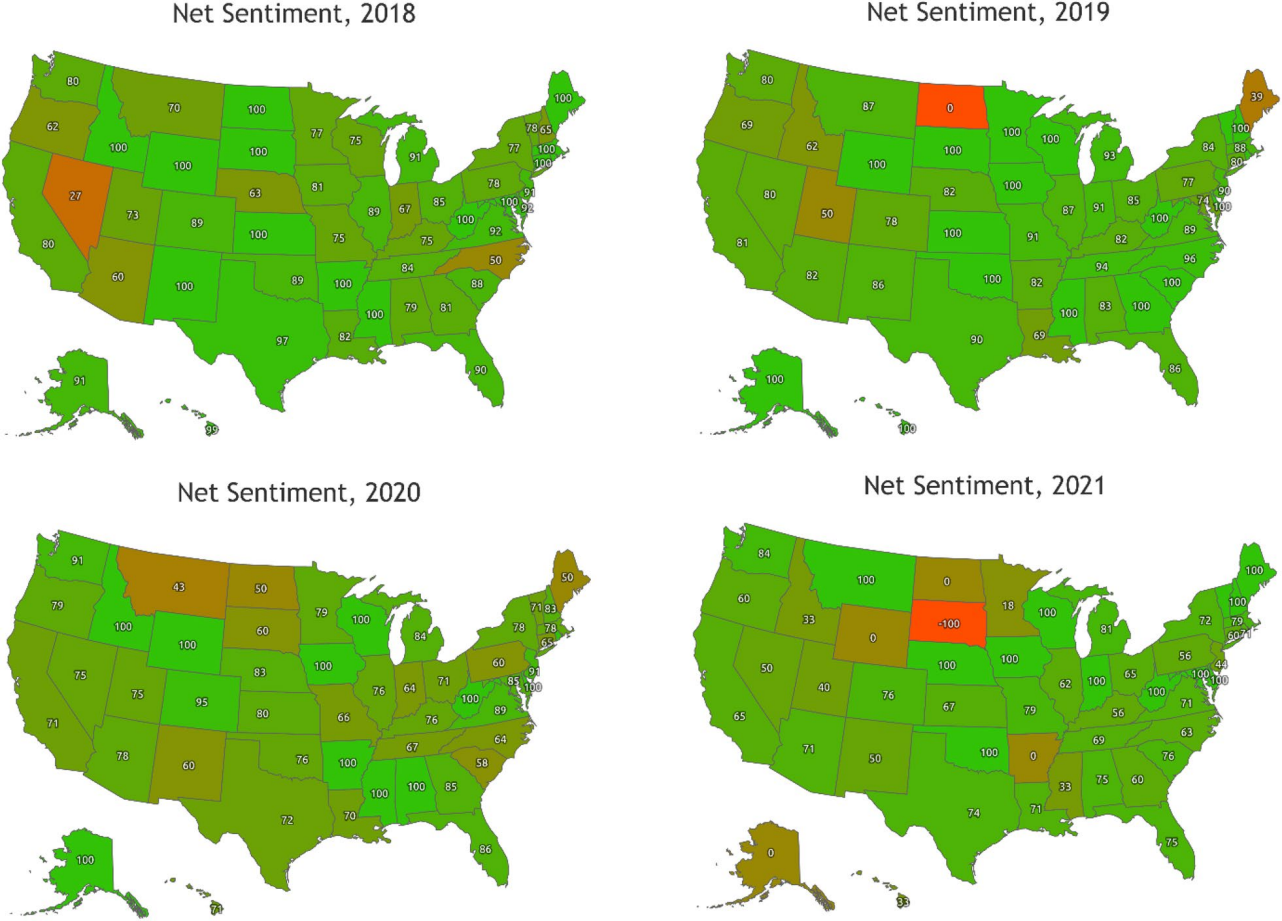
Map of the net sentiment across the US states from 2018 to 2021. Source: Authors’ collection of net sentiment from Netbase

Variations across states in local food production systems/availability, food insecurity rates, and real-time events may impact people’s perceptions of local foods. Therefore, net sentiment has been calculated for individual states in the general local food search. Figure 5 illustrates net sentiment for individual states, by year. The most popular mention in 2018 was Nevada driving its lowest positive sentiment saying that the local food plan is “not good enough” and the other one in 2021 for the negative sentiment at -100 was about a “hit and run accident” involving a local food truck [71]. In Maine in 2019, the word “staggering” emerged from a mention of a staggering increase in visitors that local food pantries struggle to keep up with. Thus, while the searches were not limited to food security terms and were instead the broad local food search parameterized, the major terms and drivers of significant sentiment changes refer to food security (or insecurity) related events varying from state to state, again reflecting the dominance of this topic within local food media and conversations.

## Conclusions

Local food has long been an interest of agricultural production systems, food retailing industries, and public policy and regulation. An abundance of previous research examines definitions of the term “local” within food and agricultural contexts and explored factors that drive consumers to shop, purchase and consume local food. In contrast to survey analyses, this study examines online and social media for unprompted conversations and posts mentioning local food through social listening analyses. Online media mentions related to local food were often about local food security and access, which was even more apparent during government employee furloughs and the more recent COVID-19 pandemic. Top terms returned in the searches conducted reference include references to local food banks/pantries and encouragements for people to donate food and money or volunteer for local food banks and pantries.

Increased volumes of mentions about local foods came immediately after peaks of confirmed COVID-19 cases, albeit dissipating with successive peaks over time. Even though mentions are usually information sources for managing through crises, social media activities on local foods appeared more likely to be active encouragement to help through donations or volunteering. Future research may further investigate why encouraging mentions decayed with successive peaks of COVID-19 confirmed cases. If this is due to pandemic fatigue but not accompanied with real-life improvement of food insecurity and local food bank/pantry situations, this may leave people in need without assistance.

## Data Availability

The data set generated and analyzed of the current study is not publicly available due to privacy/license policies associate with NetBase Solutions, Inc. That being said, the data of this study are available from the corresponding author upon reasonable request.

## Abbreviations

CDC: Center for diseases and control
CSA: Community supported agriculture
DTC: Direct-to-consumer
FDA: Food and drug administration
FSIS: Food safety and inspection service
NLP: Natural language processing
USDA: United States department of agriculture
USDA NASS: United States department of agriculture national agricultural statistics service

## Footnotes

1 Farmers markets and Community Supported Agriculture (CSA), representative indicators of DTC, in combination with other forms of DTC marketing such as On-Farm-Store or Roadside stand, DTC accounts for 35% of the total direct sales. 27% of the total direct sales comes from supplying to local retailers and another 39% from supplying to institutions (schools, colleges, and hospitals) and intermediaries (wholesalers, distributers, and processors, etc.) marketing their products locally [13]. Farmers markets continued to rise from 1,755 in 1994 and 3,706 in 2004 to 8,284 markets in 2014 nationwide [14]. Community Supported Agriculture (CSA) has also exploded in number from 2 in the mid-1980 to an estimated 12,617 farms in 2012, which is up slightly from 12,549 in 2007 [9].

2 Federal policies include Community Food Project Grants Program, WIC Famers’ Market Nutrition Program, Senior Farmers’ Market Nutrition Program, Federal State Marketing Improvement Program, National Farmers’ Market Promotion Program, Specialty Crop Block Grant Program, and Community Facilities Program [12]. The updated Farmers’ Market Promotion Program (FMPP, Sec. 10,003) expands its support to intermediated marketing channels in addition to DTC [11, 15].

3 State and local policies are closely engaged in farm-to-institution procurement, promotion of local food markets, incentives for low-income consumers to shop at farmers’ markets, and creation of State Food Policy Councils.

4 **Locavore (noun lo·ca·vore \ ˈlōkəˌvôr \)** – A person whose diet consists only or principally of locally grown or produced food (Oxford American Dictionary).
